# The Mechanism Involved in the Inhibition of Resveratrol and Genistein on the Contractility of Isolated Rat Uterus Smooth Muscle

**DOI:** 10.3390/nu16193417

**Published:** 2024-10-09

**Authors:** Qin Ma, Yudong Wang, Wei Zhang, Zhongrui Du, Zhifeng Tian, Hongfang Li

**Affiliations:** 1 Department of Physiology, College of Basic Medicine, Lanzhou University, Lanzhou 730000, China; 2 School of Pharmacy, Lanzhou University, Lanzhou 730000, China; 3 Function Laboratory in College of Basic Medicine, Lanzhou University, Lanzhou 730000, China; 4 Key Laboratory of Preclinical Study for New Drugs of Gansu Province, Lanzhou 730000, China

**Keywords:** resveratrol, genistein, smooth muscle, uterine movement, Ca^2+^ influx and release

## Abstract

Purpose: This study aimed to compare the effects of the phytoestrogens resveratrol (RES) and genistein (GEN) on the contractility of isolated uterine smooth muscle from rats, focusing on both spontaneous and stimulated contractions, and to investigate the underlying mechanisms. Methods: Uterine strips were suspended vertically in perfusion chambers containing Kreb’s solution, various concentrations of RES and GEN were added to the ex vivo uterine strips, and contractions were measured before and after incubation with RES or GEN. Results: (1) Both RES and GEN inhibited K^+^-induced contractions in a dose-dependent manner; the β/β_2_-adrenoceptor antagonist propranolol (PRO), ICI118551, the ATP-dependent K^+^ channel blocker glibenclamide (HB-419) and the NO synthase inhibitor N-nitro-L-arginine (L-NNA) diminished the inhibitory effects of RES and GEN on K^+^-induced contractions. (2) RES and GEN also dose-dependently inhibited PGF_2α_-induced uterine contractions. (3) The inhibitory effects of RES and GEN were observed in spontaneous contractile activities as well; PRO, ICI118551, HB-419 and L-NNA attenuated the inhibitory effects of RES and GEN on the spontaneous contractions of isolated uterine muscle strips. (4) RES and GEN significantly decreased the cumulative concentration response of Ca^2+^ and shifted the Ca^2+^ cumulative concentration–response curves to the right in high-K^+^ Ca^2+^-free Kreb’s solution. (5) RES and GEN markedly reduced the first phasic contraction induced by oxytocin, acetylcholine, and prostaglandin F_2α_ but did not alter the second phasic contraction caused by CaCl_2_ in Ca^2+^-free Kreb’s solution. Conclusions: RES and GEN can directly inhibit both spontaneous and activated contractions of isolated uterine smooth muscle. The mechanisms underlying the inhibitory effects of RES and GEN likely involve β adrenergic receptor activation, reduced Ca^2+^ influx and release, the activation of ATP-dependent K^+^ channels and increased NO production.

## 1. Introduction

Uterine contractility is indispensable for the uterus to work properly. Research has demonstrated that adequate uterine contractility is necessary for the transportation of gametes and embryos, as well as for embryo implantation [1]. Estrogen plays a crucial role in the synthesis of the contractile proteins required for uterine contractility [2]. Additionally, it can up-regulate the expression of endometrial oxytocin and oxytocin receptor mRNA [3].

**Figure 1:**
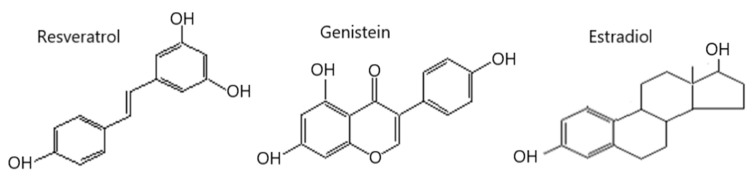
Structural comparison of resveratrol and genistein with estradiol.

Phytoestrogens are natural compounds derived from plants, found in various foods such as soy products, bean sprouts, clover, flax seeds, grains and grapes, as well as several medicinal plants, including *Syringa villosa*, *Astragalus membranaceus*, *Radix puerariae*, *Humulus lupulus*, *Verbena*, etc. [4,5,6]. These compounds share structural similarities with mammalian estrogens, which allows them to bind with estrogen receptors [6,7]. Among the phytoestrogens, resveratrol (RES) and genistein (GEN) have garnered significant scientific interest in recent years due to their potential health effects [8,9]. RES, a polyphenolic stilbene, closely resembles natural estradiol (Figure 1), particularly in its similarity to diethylstilbestrol, and acts as a mixed estrogen receptor agonist. It exhibits affinity for both estrogen receptors α and β (ERα and ERβ) [10] and can also stimulate membrane-bound ER [11]. The direct binding of RES to nuclear ER activates its genomic activity, which was the first reported pharmacological action; however, its interaction with membrane-bound ER is associated with rapid non-genomic estrogenic activities [11]. GEN is a natural compound belonging to the flavonoid class (Figure 1) that can bind to both the classic ERα found in reproductive organs and the novel ERβ present in the vasculature, albeit with a lower affinity than that of estradiol [12]. Notably, GEN exhibits a 20-fold greater affinity for ERβ compared to ERα, and it is believed that its adverse effects are associated with its binding to ERα, while its beneficial effects are linked to ERβ [13]. The use of phytoestrogens such as RES and GEN as natural sources of estrogen has gained rapid acceptance for various therapeutic and preventive applications, including those for obesity [14,15], osteoporosis [16,17], climacteric symptoms [18], cardiovascular disease [19,20,21] and breast cancer prevention [22]. Furthermore, phytoestrogen-rich legumes are of high economic value as pasture crops in sheep and cattle production enterprises; thus, due to their role in the reproductive system, proactive management strategies are needed to mitigate the risk of potential fertility loss due to the use of legumes as a feed for grazing livestock [23].

Several studies have suggested that RES and GEN can reduce vascular contractile responses and promote the relaxation of vascular smooth muscles through a Ca^2+^ antagonistic property [19,20,24]. RES has been shown to inhibit uterine contractions by blocking the influx of external Ca^2+^ [25] and has potentially beneficial effects for ameliorating ovarian and testis function [26]. While GEN can inhibit the smooth muscle contractions of isolated rat prostate glands [27], its effects on mammalian reproductive capacity are controversial and are dependent on the dose, the species, the gender and the route and time of administration [12]. Additionally, both RES and GEN can inhibit spontaneous and induced contractions of gallbladder smooth muscle [28]. Electrophysiological studies indicated that GEN reversibly inhibits the L-type calcium current in isolated guinea pig ventricular myocytes in a concentration-dependent manner [29]. Therefore, the biological actions of RES and GEN are highly beneficial for various therapeutic and preventive purposes, suggesting their potential as natural substitutes for estrogen therapy. Despite the growing interest in the effects of phytoestrogens on the reproductive system, little is known about the impact of RES and GEN on uterine movement. This study aims to compare the effects of RES and GEN on the contraction of rat myometrium, including both spontaneous and stimulated contractions, and to investigate the underlying mechanisms.

## 2. Materials and Methods

### 2.1. Drugs

Genistein (GEN), resveratrol (RES), propranolol (PRO), erythro-DL-1-(7-Methylindan-4-yloxy)-3-isopropylamino-2-butanol (ICI 118551), oxytocin (OXY), 9-(Tetrahydro-2′-furyl)adenine (SQ 22536), N-nitro-L-arginine (L-NNA), acetylcholine (ACH), indomethacin (IND) (Sigma, Chemical, St. Louis, MO, USA); glibenclamide (HB-419), (N-[2-(p-Bromocinnamylamino)ethyl]-5-isoquinolinesulfonamide (H-89), 7a,17b-[9-[(4,4,5,5-Pentafluoropentyl)sulfinyl]nonyl]estra-1,3,5(10)-triene-3,17-diol (ICI 182780), prostaglandin F_2α_ (PGF_2α_) (Tocris Cookson Inc., Bristol, UK); potassium bisperoxo (1,10 phenanthro line) oxovanadate BPV (phen) (Alexis Biochemicals, San Diego, CA, USA). The solutions of genistein, resveratrol, propranolol, ICI118551, HB-419, L-NNA, ICI182780, H-89 and PGF_2α_ were prepared with dimethyl sulphoxide (DMSO), while the others were prepared with distilled water.

### 2.2. Animal and Uterine Strip Preparation

All animal experiments were performed in accordance with the procedures described in the Guide for the Care and Use of Laboratory Animals of the National Institute of Health, and the procedures performed were consistent with institutional guidelines [30]. Adult female Sprague Dawley rats (SD rats, weighing 230–250 g, n = 85, provided by the experimental animal center of Lanzhou University) were used in this study. The rats were housed in a temperature-controlled room (23 ± 2 °C) with native illumination for 14 h/d, with food and water provided freely before their sacrifice in the experiment; all the animals were served with a phytoestrogen-free basic chow.

The rats were fasted overnight (12 h) with free access to water. The experimental rats were anesthetized with 0.3% sodium pentobarbitone administered intramuscularly before cervical dislocation [27], and the whole uteri were quickly removed and placed in a culture dish filled with Kreb’s solution containing the following (mmol/L): NaCl 120, KCl 5.9, NaH_2_PO_4_ 1.2, MgCl_2_ 1.2, NaHCO_3_ 15.4, CaCl_2_ 2.5 and glucose 11.5, buffered at pH 7.4. Uterine smooth muscle strips (5 mm × 10 mm) were prepared [25] and tied to the ventilated hooks through the thread. After that, the muscle strips were hung longitudinally in separate 5 mL perfusion chambers containing (37 ± 0.5 °C) Kreb’s solution, continuously ventilated with a gas mixture of 95% O_2_ and 5% CO_2_. The muscle strips were equilibrated for at least 30 min with a preload of 1.5 g, and the solution was changed every 20 min. After equilibration, the uterine strips were treated with RES and GEN alone or combined with various drugs (10^−6^ mol/L PGF_2α_, 10^−5^ mol/L PRO, 10^−6^ mol/L ICI118551, 10^−5^ mol/L HB-419, 10^−5^ mol/L L-NNA, 10^−5^ mol/L ICI182780, 10^−6^ mol/L BPV, 10^−6^ mol/L H-89, 10^−6^ mol/L SQ22536, 10^−5^ mol/L IND, 0.4 IU/L OXY, 5 × 10^−5^ mol/L ACH or 40/80 mmol/L KCl) to observe the change in the uterine contraction. The spontaneous and stimulated contractile activities of the uterine smooth muscles were measured with force transducers and recorded with the BL-420E^+^ biological signal acquisition and processing system (TME, Chendu, China) by microcomputer.

### 2.3. Experimental Protocols

To explore the effects of RES and GEN on the basal contractile activities of the uterus, different concentrations (0.1, 0.5, 1.0, 5.0, 10.0, 50.0 or 100.0 μmol/L) of RES or GEN, or the same dose of solvent DMSO (control), were added to the perfusion chamber every 5 min [28]. The different drugs were administered after the contracting waves had reached equilibrium. The different drugs as mentioned above were added into the perfusion chambers 5 min before the administration of RES or GEN to explore whether their actions were related to the β adrenergic receptor, estrogen receptors, ATP-dependent K^+^ channel, voltage-operated Ca^2+^ channels (VOCs) in uterine smooth muscle, Ca^2+^ release from the sarcoplasmic reticulum, receptor-operated channels (ROCs), cyclic adenosine 5′-monophosphate (cAMP) synthase, phosphotyrosine phosphatase, protein kinase A, endogenous prostaglandin synthesis or NO production.

To observe the influence of RES or GEN on the contractile response stimulated by high K^+^ (40 mmol/L KCl) or PGF_2α_ (10^−6^ mol/L), KCl was added into the perfusion chambers when there were nearly no rhythmical waves of uterine contraction, only a straight line. Tonic contraction of the uterine smooth muscles was induced by KCl to maximal contraction, and then RES or GEN (0.1–100.0 μmol/L) was administrated to the uterine smooth muscle strips through cumulative dosage. The effects of RES or GEN on the KCl-induced contraction were then observed. Similarly, PGF_2α_ was added into the perfusion chambers, the contractile activities of uterine smooth muscle were activated and enhanced, and RES and GEN (0.1–100.0 μmol/L) were then added cumulatively after the PGF_2α_-induced contractions had stabilized.

To evaluate the possible effect of RES or GEN on calcium influx through VOCs, the strips were incubated in Kreb’s solution without Ca^2+^ but containing 0.01 mmol/L egtazic acid (EGTA) for 30 min and then treated with KCl (80 mmol/L). When the contracting response reached its peak and stabilized, CaCl_2_ solution (10^−5^, 10^−4.5^, 10^−4^, 10^−3.5^, 10^−3^, 10^−2.5^ or 10^−2^ mol/L) was added into the perfusion chamber to establish a dose-dependent contraction curve (control). Then, the strips were rinsed with Kreb’s solution to return their tension to the baseline level; after that, the Kreb’s solution was replaced with calcium-free Kreb’s solution for another 30 min. The strips were then treated with KCl again after incubation with RES (50 μmol/L) or GEN (50 μmol/L) for 8–10 min, and the CaCl_2_ response contraction curves were obtained again.

In another experiment, in order to estimate the prospective effects of RES or GEN on ROCs or Ca^2+^ release, myometrial strips were incubated in Kreb’s solution without Ca^2+^ but containing 0.01 mmol/L egtazic acid (EGTA) for 30 min, then treated with OXY (0.4 IU/L), ACH (5 × 10^−5^ mol/L) or PGF_2α_ (10^−5^ mol/L). When the contraction reached a peak, CaCl_2_ was added immediately, and a further contraction was obtained (control). After washout and equilibration, RES or GEN (50 μmol/L) was added into the chambers for 8–10 min before all the procedures mentioned above were repeated, and then the response contraction curve was obtained.

### 2.4. Statistical Analysis

All results are expressed as the mean ± SEM. “n” refers to the number of rats. For KCl (40 mmol/L)-induced contractions or basal contractions, data are expressed as the percentage decrease in the tension in the muscle strip, the contraction wave amplitude, and the frequency after the addition of RES and GEN, which were recorded as zero before the dosing. In the CaCl_2_ concentration–response curves, the percentage of the maximal CaCl_2_ contraction, E_max_, EC_50_ and its pD_2_ value were calculated by referring to a previously published paper [19,20]. Statistical analysis was performed by using Student’s *t*-test and a one-way ANOVA with SPSS version 21.0 software. *p* < 0.05 was considered significant.

## 3. Results

### 3.1. Influences of Resveratrol and Genistein on KCl-Induced Contractions

**Figure 2:**
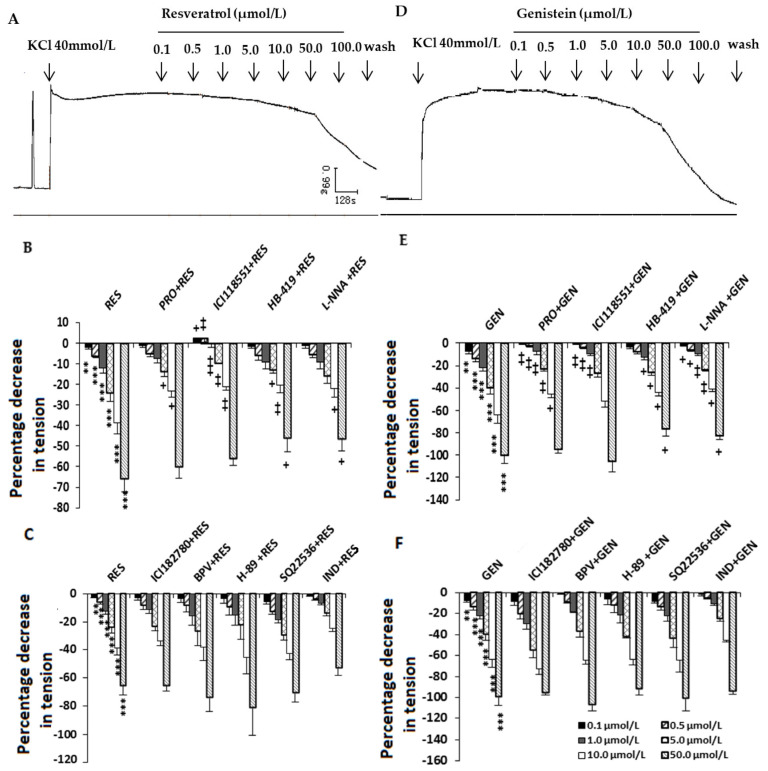
Effects of resveratrol (RES) and genistein (GEN) on KCl-induced uterine contractions. Typical recording of the effects of RES (**A**) and GEN (**D**) on KCl-induced uterine contractions and effects of different antagonists on the inhibition induced by RES (**B**,**C**) and GEN (**E**,**F**) in KCl-precontracted strips. β adrenoceptor antagonist propranolol (PRO) and ICI118551; ATP-dependent K^+^ channel blocker glibenclamide (HB-419); NO synthase inhibitor N-nitro-L-arginine (L-NNA); estrogen receptor antagonist ICI182780; phosphotyrosine phosphatase inhibitor (BPV); protein kinase A inhibitor (H-89); cAMP synthase inhibitor (SQ22536); prostaglandin synthase inhibitor indomethacin (IND). ** *p* < 0.01; *** *p* < 0.001; vs. control group (the value was set as zero). ^+^ *p* < 0.05; ^++^ *p* < 0.01; ^+++^ *p* < 0.001; vs. RES or GEN group.

The present studies showed that high K^+^ concentrations could induce the tonic contraction of the uterine smooth muscle strips (Figure 2A,D). RES and GEN (0.1–100.0 μmol/L) caused an obvious dose-dependent inhibition of KCl-induced contractions (RES: Figure 2A,B, n = 12; GEN: Figure 2D,E, n = 12).

### 3.2. Effects of Different Blockers on the Inhibition Induced by Resveratrol and Genistein in KCl-Precontracted Uterine Muscle Strips

In the tonic contraction stimulated by KCl (40 mmol/L), when the uterine smooth muscles were previously treated with the β adrenoceptor antagonist PRO (RES: n = 12; GEN: n = 10), the selective β_2_ adrenoceptor antagonist ICI118551 (RES: n = 6; GEN: n = 5), the ATP-dependent K^+^ channel blocker HB-419 (RES: n = 9; GEN: n = 6) or the NO synthase inhibitor L-NNA (RES: n = 11; GEN: n = 10), the inhibitory effects induced by RES (Figure 2B) and GEN (Figure 2E) were significantly reduced; however, the estrogen receptor antagonist ICI182780 (RES: n = 7; GEN: n = 5), the potent phosphotyrosine phosphatase inhibitor BPV (RES: n = 5; GEN: n = 3), the inhibitor of cAMP synthase SQ22536 (RES: n = 7; GEN: n = 5), the inhibitor of specific protein kinase A H-89 (RES: n = 4; GEN: n = 3) and the prostaglandin synthase inhibitor IND (RES: n = 9; GEN: n = 6) did not alter the inhibition by RES (Figure 2C) or GEN (Figure 2F).

### 3.3. Effects of Resveratrol and Genistein on PGF_2α_-Induced Uterine Contractions

**Figure 3:**
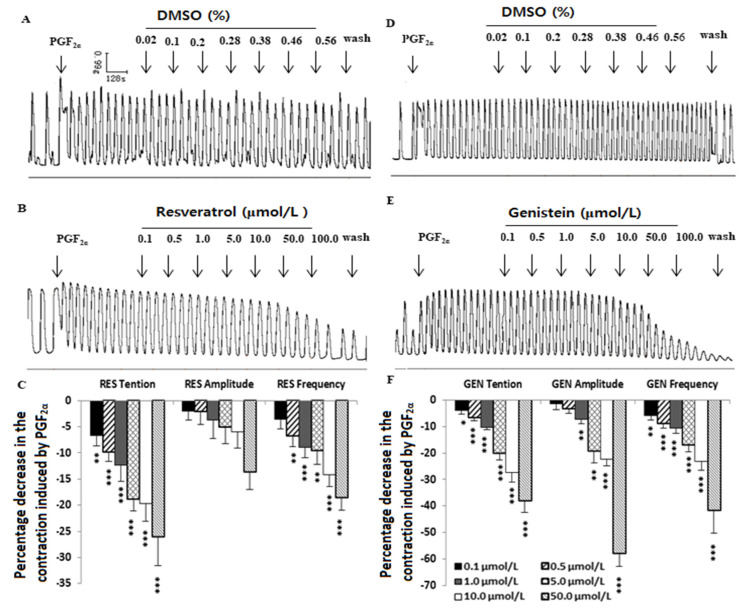
Effects of resveratrol (RES) and genistein (GEN) on PGF_2α_-induced uterine contractions. Typical recordings of the effects of DMSO (**A**,**D**) and RES or GEN (**B**,**E**) on PGF_2α_-induced uterine contractions; the tension, mean amplitude and frequency of the uterine contractions are shown in (**C**,**F**). * *p* < 0.05; ** *p* < 0.01; *** *p* < 0.001 vs. control group (the value was set as zero).

PGF_2α_ (10^−6^ mol/L) increased the rhythmic contraction of the uterine muscle strips, but compared with DMSO (Figure 3A,D), RES and GEN (0.1–100.0 μmol/L) dose-dependently inhibited the PGF_2α_-induced contraction (RES: n = 12, Figure 3B,C; GEN: n = 10, Figure 3E,F) and markedly decreased its tension, mean amplitude and mean frequency.

### 3.4. Influences of Resveratrol and Genistein on Uterine Myogenic Spontaneous Contractions

**Figure 4:**
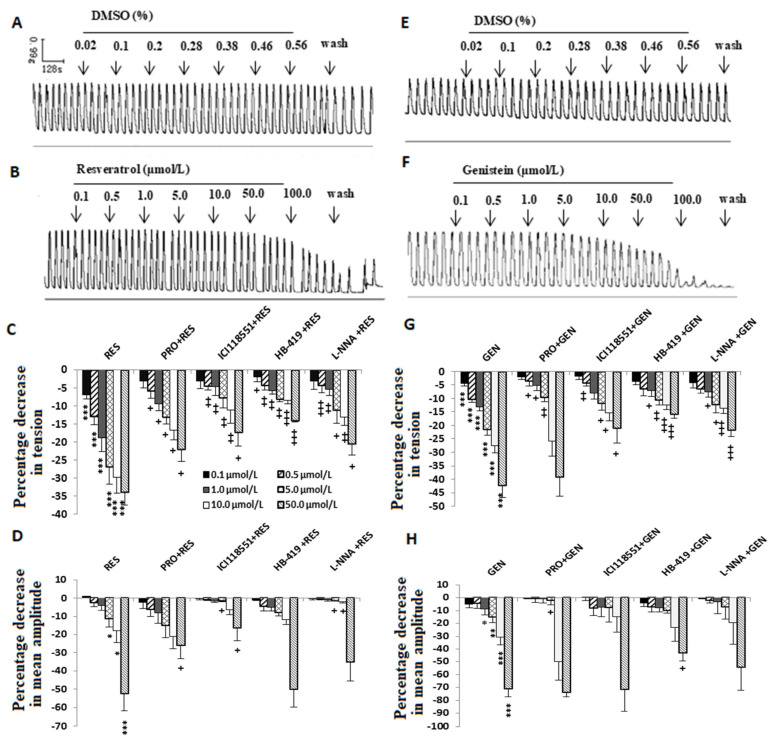
Effects of resveratrol (RES) and genistein (GEN) on spontaneous phasic contractions. Typical recordings after administration of the solvent DMSO (**A**,**E**), RES (**B**) or GEN (**F**); the tension (**C**,**G**) and the mean amplitude (**D**,**H**) of the uterine contractions are shown after treatments with different drugs such as β adrenoceptor antagonist propranolol (PRO) and ICI118551, ATP-dependent K^+^ channel blocker glibenclamide (HB-419) and NO synthase inhibitor N-nitro-L-arginine (L-NNA). * *p* < 0.05; ** *p* < 0.01; *** *p* < 0.001; vs. control group (the value is set as zero). ^+^ *p* < 0.05; ^++^ *p* < 0.01; ^+++^ *p* < 0.001; vs. RES or GEN group.

Compared with the solvent DMSO (Figure 4A,E), RES (Figure 3B) and GEN (Figure 4F) had an inhibitory effect on the spontaneous contraction of uterine smooth muscle. That is to say, in the muscle strips with myogenic spontaneous contractions, RES (Figure 3C,D) and GEN (Figure 4G,H) dose-dependently reduced their contractile activities; they not only produced an obvious reduction in resting tone (Figure 4C,G) but also markedly decreased the mean amplitude of the rhythmic contractions (Figure 4D,H).

### 3.5. Influences of Different Blockers on the Inhibitory Effects Induced by Resveratrol and Genistein on Uterine Spontaneous Contractions

In terms of the spontaneous contractile activity of the uterine smooth muscle, PRO (RES: n = 12; GEN: n = 4), ICI118551 (RES: n = 3; GEN: n = 4), HB-419 (RES: n = 5; GEN: n = 6) and L-NNA (RES: n = 6; GEN: n = 6) markedly attenuated the inhibitory effects caused by RES and GEN (Figure 4C,D,G,H).

### 3.6. Effects of Resveratrol and Genistein on Uterine Ca^2+^-Dependent Contractions

**Figure 5:**
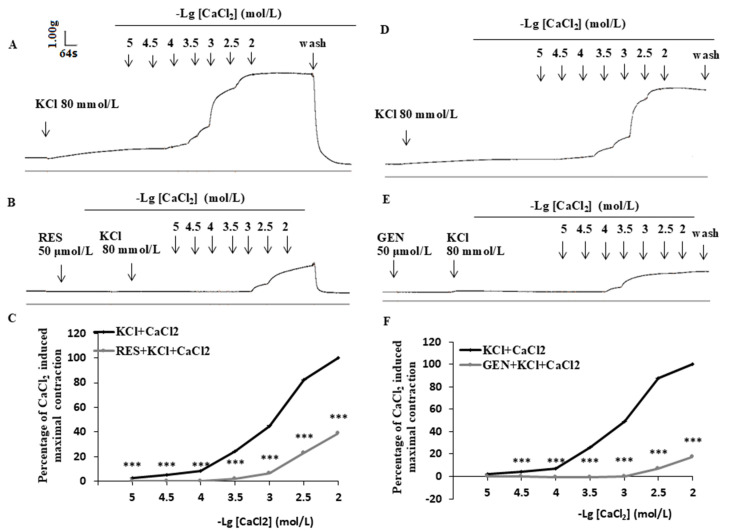
Effects of resveratrol (RES) and genistein (GEN) on CaCl_2_ dose-dependent contraction curves. Traces of CaCl_2_-induced contraction of uterine smooth muscle in Ca^2+^-free Kreb’s solution in the absence and presence of RES (**A**,**B**) or GEN (**D**,**E**). Line plots show the effects of RES ((**C**), 50 μmol/L) and GEN ((**F**), 50 μmol/L) on CaCl_2_ dose-dependent contraction curves in isolated uterine smooth muscle strips. *** *p* < 0.001; vs. KCl + CaCl_2_ group.

When CaCl_2_ solution (10^−5^–10^−2^ mol/L) was added cumulatively to the perfusion chamber, a concentration-dependent contraction reaction appeared; RES and GEN (50 μmol/L) markedly decreased the Ca^2+^ cumulative concentration response and shifted the Ca^2+^ concentration-dependent response curves rightward in high-K^+^ Ca^2+^-free Kreb’s solution (Figure 5A,B,D,E). The maximal contraction of the muscle strips was inhibited by RES (n = 11, Figure 5C) and GEN (n = 10, Figure 5F). The main pharmacological parameters before and after the administration of RES and GEN were as follows: con_RES_: Emax = 110.99 ± 5.93, EC_50_ = 1.430 × 10^−3^ mmol/L, pD_2_ = 2.845 ± 0.08; RES_50μmol/L_: Emax = 45.17 ± 6.45, EC_50_ = 3.148 × 10^−3^ mmol/L, pD_2_ = 2.502 ± 0.13 (all *p* < 0.01 vs. con_RES_); con_GEN_: Emax = 107.26 ± 3.03, EC_50_ = 1.117 × 10^−3^ mmol/L, pD_2_ = 2.952 ± 0.05; GEN_50μmol/L_: Emax = 19.09 ± 8.47, EC_50_ = 3.635 × 10^−3^ mmol/L, pD_2_ = 2.440 ± 0.31 (all *p* < 0.01 vs. con_GEN_).

### 3.7. Influences of Resveratrol and Genistein on Biphasic Contraction Caused by Oxytocin, Acetylcholine, Prostaglandin F_2α_ and CaCl_2_

**Figure 6:**
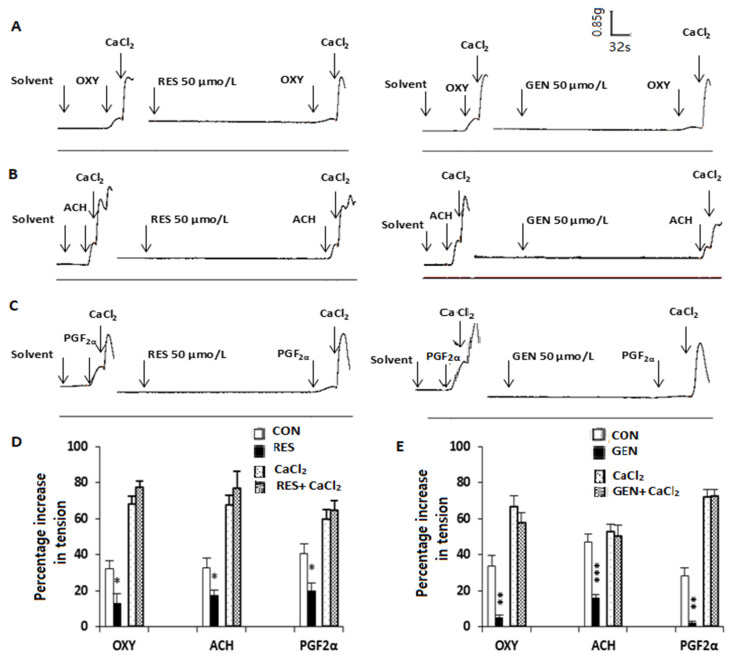
Effects of resveratrol (RES) and genistein (GEN) on biphasic contraction in isolated uterine smooth muscle. Traces of contractions induced by oxytocin (OXY, (**A)**), acetylcholine (ACH, (**B**)), prostaglandin F_2α_ (PGF_2α,_ (**C**)) and CaCl_2_ in uterine smooth muscle strips in Ca^2+^-free Kreb’s solution in the absence and presence of RES and GEN. Bar charts show the effects of RES (**D**) or GEN (**E**) on the OXY-, ACH-, PGF_2α_- and CaCl_2_-induced uterine contraction. * *p* < 0.05; ** *p* < 0.01; *** *p* < 0.001; vs. solvent control group.

In calcium-free Kreb’s solution, OXY (0.4 IU/L, Figure 6A), ACH (5 × 10^−5^ mol/L, Figure 6B) and PGF_2α_ (10^−5^ mol/L, Figure 6C) caused a transient contraction. When this contraction reached its peak, the administration of CaCl_2_ 20 mmol/L induced a further contractile response. RES (50 μmol/L, Figure 6D) and GEN (50 μmol/L, Figure 6E) markedly reduced the first contraction induced by OXY, ACH and PGF_2α_ but did not change the second contraction caused by CaCl_2_.

## 4. Discussion

The non-pregnant uterus, whether in rodents or humans, is not a quiescent organ; it exhibits wave-like activity throughout the whole menstrual cycle [31,32,33]. Studies have shown that uterine contractility seems to be related to oocyte migration through the oviduct, sperm movement, embryonic transport from the fallopian tube to the uterine cavity and possibly embryo implantation itself [33]. Abnormal uterine contractility (dyskinesia) may cause pelvic pain (dysmenorrhea), retrograde bleeding with dysmenorrhea, endometriosis, ectopic pregnancies, miscarriages and preterm labor [34,35,36]; therefore, further elucidating the characteristics of uterine movement may provide an opportunity to correct abnormal activity wave patterns, improve the rate of embryo implantation and alleviate the symptoms of dysmenorrhea and endometriosis, thereby enhancing pregnancy rates and animal fertility.

Previous studies demonstrated that the concentration of Ca^2+^ is an essential factor in the contraction of uterine smooth muscle, and it can be regulated by two important Ca^2+^ channels: receptor-operated channels (ROCs) and voltage-operated calcium channels (VOCs) [37,38]. When the extracellular K^+^ concentration is increased, VOCs are activated by depolarization of the plasma membrane, the L-type VOCs are opened, and Ca^2+^ influx through the VOCs is increased. In the present study, it appears that KCl (40 mmol/L) caused tonic contraction, but this uterine contraction was decreased after RES or GEN was cumulatively administered in normal Kreb’s solution. It is obvious that RES and GEN have inhibitory effects on KCl-induced contractions of uterine smooth muscles, and this effect may be regulated by the influx of extracellular Ca^2+^ through the VOCs. In calcium-free high-K^+^ Kreb’s solution, RES and GEN observably suppressed CaCl_2_-induced contraction; either of them could apparently shift the CaCl_2_ cumulative concentration–response curves rightward. It is obvious that uterine contractility is essential for uterine excitability and motility, as well as successful labor; in turn, it relies on Ca^2+^ influx through VOCs on the cell membrane of uterine smooth muscle [39,40]. Taken together, the results suggest that a decrease in extracellular Ca^2+^ influx through the VOCs plays a crucial role in the inhibitory effects of RES and GEN. In the ROCs or referring to Ca^2+^ release, when OXY, ACH and PGF_2α_ activate the cellular membrane G-protein-coupled receptors, the cytoplasmic concentration of Ca^2+^ is increased by enhanced ROC Ca^2+^ influx and sarcoplasmic reticulum Ca^2+^ release [37]. In our experiment, RES and GEN reduced the first phasic contraction induced by OXY, ACH and PGF_2α_ but did not change the second phasic contraction caused by CaCl_2_. These results demonstrate that the inhibition of Ca^2+^ release probably mediates the inhibitory effects of RES and GEN on the spontaneous and activated contractile activities of uterine smooth muscle, while the inhibitory effects of RES and GEN may not be related to Ca^2+^ influx through the ROCs.

Propranolol and ICI 118551, selective β/β_2_ adrenergic receptor antagonists [41], were used in the present experiment to assess whether the inhibitory effects of RES and GEN on the uterine contractile activity were relevant to the β-adrenoceptor receptor, especially the β_2_ adrenoceptor. The data from the present study suggest that the action of RES or GEN in suppressing the contractile activity of uterine smooth muscle was markedly changed by the β-adrenoceptor antagonists propranolol and ICI 118551; thus, the inhibitory effects of RES and GEN probably have a relationship with the activation of β-adrenoceptor receptors.

Studies have previously shown that glibenclamide (HB-419) is a selective antagonist of K^+^ channel openers in vitro [42]. HB-419 has been demonstrated to inhibit the opening of ATP-dependent K^+^ channels in the pancreas, the heart and the mesenteric artery. In our present study, the inhibitory effects of RES and GEN were reduced by HB-419 in both the spontaneous and the KCl-induced contractile activity of uterine smooth muscle; the ability of HB-419 to antagonize the uterine effects of RES and GEN supports the idea that these drugs act by ATP-dependent K^+^ channel opening in the reproductive system in vitro.

A large number of studies have prompted assessments of the role of NO in the regulation of uterine contractility and suggested that NO donors are, in fact, capable of altering uterine contractility [43,44,45]. A study on pregnant rats reported that an L-arginine–NO–guanosine cyclic monophosphate (cGMP) system exists in the uterus, which can inhibit uterine contractility [46]. The substrate and donors of NO were shown to relax uterine tissues, and these effects were reversed by inhibitors of the NO-cGMP pathway [47]. In the present study, the inhibitory effects of RES and GEN on spontaneous contractions and KCl-induced contractions in uterine smooth muscle were changed by adding L-NNA; our data imply that the inhibitory effects of RES and GEN are related to NO production.

The uterus is an important target organ for steroid hormones whose effects are mediated by estrogen receptors (ERs). There are two different types of ERs on uterus tissues: the classical type comprises the nuclear ERα and ERβ, which mediate gene expression through binding to estrogen receptor elements in the promotor and regulatory regions of the target genes; the other type of estrogen receptor is the membrane estrogen receptor, a 7-transmembrane spanning G-protein-coupled receptor, also called G-protein-coupled receptor 30 (GPR30), which mediates rapid cellular effects and is structurally and genetically unrelated to ERα and ERβ and expressed independently [48]. It was reported that the distribution and regulation of ERα and ERβ differ in the different compartments of the rat uterus. ERα is the dominating subtype in the rat uterus and is mainly expressed in the glandular and luminal epithelium; in contrast, ERβ is found to be mainly expressed in the uterine vasculature [12,49,50]. Recently, it was found that GPR30 was expressed and functional in rat myometrium [48] and that the activation of GPR30 produces depolarization, elevates [Ca^2+^] (i) and increases contractility in myometrial cells [51]. Another paper reported that RES can inhibit Kv2.2 currents through the estrogen receptor GPR30-mediated PKC pathway [52]. In order to explore the relationship between the influences of RES and GEN on uterine smooth muscle contraction and the estrogen receptors ERα and ERβ, ICI 182,780, a non-selective estrogen receptor antagonist (blocking both ERα and ERβ) [53], was used in this experiment. The inhibitory inotropic action induced by RES and GEN showed no obvious change, suggesting that the inhibitory actions of RES and GEN on uterine smooth muscle contraction are not mediated by ERs.

RES and GEN possess the inhibitory properties of tyrosine kinase, and evidence indicates that tyrosine kinase activity plays an important role in the contractility of smooth muscle [54,55,56]. In our study, a potent protein tyrosine phosphatase inhibitor, BPV (phen), which can increase protein tyrosine phosphorylation, did not attenuate the inhibitory effects of RES and GEN. This suggests that the suppression of tyrosine kinase is not related to the inhibition induced by RES or GEN in uterine movement.

Previous studies [57,58] have reported that activating protein kinase A by elevating the cyclic adenosine monophosphate (cAMP) level can inhibit myometrial contractility, and an enhancement of sarcoplasmic reticulum Ca^2+^ cycling is associated with an increase in the cAMP level. In the present work, we demonstrated that the adenylyl cyclase inhibitor SQ22536 did not abolish the effects of RES and GEN on uterine smooth muscle. This suggests that the effects of RES and GEN are independent of the activation of adenylyl cyclase and cAMP. We also demonstrated that H-89, an inhibitor of specific protein kinase A, could not impair the inhibitory effects of RES and GEN on uterine contractile activity. These findings suggest that the effects of RES and GEN are not mediated by cAMP signals.

PGF_2α_ can increase the contraction of uterine smooth muscle or small endometrial blood vessels, causing uterine tissue ischemia and endometrial disintegration, resulting in bleeding and pain symptomatic of dysmenorrhea [59,60,61]. Previous papers reported that the PGF_2α_ levels are elevated in women with primary dysmenorrhea as compared with their asymptomatic counterparts. Still, some studies have shown that dysmenorrhea can also lead to increased PG (PGE_2_ and PGF_2α_) production, so there is a vicious cycle: the strong contraction of the blood vessels and myometrium induced by PGF_2α_ can aggravate dysmenorrhea [59,60,61]. Though PGs are implicated in dysmenorrhea, this condition is considered to be directly related to elevated PGF_2α_ levels, and it is well established that PGF_2α_ increases the concentration of Ca^2+^ and then stimulates uterine contraction [62,63]. In the present experiment, RES and GEN markedly suppressed exogenous PGF_2α_-induced uterine contractions; however, indomethacin, an inhibitor of endogenous prostaglandin synthesis, did not change the inhibitory effect induced by RES and GEN. All this evidence indicates that RES and GEN probably do not affect endogenous prostaglandin synthesis but can modulate uterus contractility by suppressing PGF_2α_-induced uterine contractions; therefore, RES and GEN may have the potential for use in the treatment or improvement of dysmenorrhea.

The phytoestrogens RES and GEN have many beneficial effects on human health, but there have been reports that they also show some adverse reproductive effects. For example, in three women, abnormal uterine bleeding was found to be related to a high intake of soy products [7]; neonatal GEN exposure also causes implantation defects, and the dietary phytoestrogen genistein impairs fertility and persistently alters the transcriptome in the oviduct and uterus of rodents [64,65]. It has been reported that this phenomenon is related to the effect of phytoestrogens on the growth of the endometrium and the disruption of glucocorticoid signaling [64,65]. Based on the results of this study, it is possible that the gamete/embryo cannot be transported and implant in a timely and appropriate manner because of the inhibition of uterine movement induced by phytoestrogens, contributing to the above reproductive abnormalities. Although the present study clarified that these compounds have obvious inhibitory effects on isolated uterine smooth muscle, further studies are needed to confirm their effects on uterus movement in vivo, the administration route, the metabolic fate of these compounds and the drug dose relationship in the future.

## 5. Conclusions

In summary, our findings suggest that the phytoestrogens RES and GEN can directly inhibit the spontaneous and activated contraction of uterine smooth muscle. The mechanisms underlying the inhibitory effects of RES and GEN are probably due to β adrenergic receptor activation, Ca^2+^ influx inhibition through voltage-operated calcium channels, Ca^2+^ release reduction from the sarcoplasmic reticulum, ATP-dependent K^+^ channel activation and NO production. Therefore, RES and GEN are probably beneficial for the alleviation of dysmenorrhea caused by PGF2α and can also affect gamete/embryo transportation and proper implantation. These results have important theoretical implications for reproductive health and dietary hygiene.

## Data Availability

The original contributions presented in the study are included in the article; further inquiries can be directed to the corresponding authors.

## Abbreviations

PE: phytoestrogen; GEN, genistein; RES, resveratrol; PRO, propranolol; ICI 118551, erythro-DL-1-(7-Methylindan-4-yloxy)-3-isopropylamino-2-butanol; OXY, oxytocin; SQ 22536, 9-(Tetrahydro-2′-furyl)adenine; L-NNA, N-nitro-L-arginine; ACH, acetylcholine; IND, indomethacin; HB-419, glibenclamide; H-89, N-[2-(p-Bromocinnamylamino)ethyl]-5-isoquinolinesulfonamide; TAM, tamoxifen; ICI 182780, 7a,17b-[9-[(4,4,5,5-Pentafluoropentyl)sulfinyl]nonyl]estra-1,3,5(10)-triene-3,17-diol); PGF2α, prostaglandin F2α; BPV, potassium bisperoxo (1,10 phenanthro line) oxovanadate BPV (phen); DMSO, dimethyl sulphoxide; ROCs, receptor-operated channels; VOCs, voltage-operated calcium channels; ANOVA, one-way analysis of variance.
